# Pathbreak: A Biodiversity-Food-Governance Game for experiential reimagination of social-environmental futures

**DOI:** 10.1038/s44185-026-00160-7

**Published:** 2026-10-08

**Authors:** Ilkhom Soliev, Michał Pająk, Maryna Bykova, Marco A. Janssen

**Affiliations:** 1 Department of Environmental Sociology, Institute of Sociology, Faculty of Philosophy I: Social Sciences and Historical Cultural Studies, Martin Luther University Halle-Wittenberg, Halle (Saale), Germany; 2 Social Learning and Environmental Governance (SLEG) Lab, Centre for Interdisciplinary Regional Studies (ZIRS), Martin Luther University Halle-Wittenberg, Halle (Saale), Germany; 3 German Centre for Integrative Biodiversity Research (iDiv), Leipzig, Germany; 4 Wroclaw University of Economics and Business, Wrocław, Poland; 5 Culture Goes Europe (CGE) Erfurt e.V., Erfurt, Germany; 6 Center for Behavior, Institutions and the Environment (CBIE), Arizona State University, Tempe, AZ USA

**Keywords:** Ecology, Ecology, Environmental social sciences, Environmental studies, Geography, Geography, Social sciences

## Abstract

Social dilemmas are central to addressing biodiversity loss, yet tools to make them tangible remain critically scarce. Pathbreak: A Biodiversity-Food-Governance Game provides an engaging and hands-on experience to explore social dilemmas between short- and long-term, individual and collective interests, alongside those pertaining to trust, elections, democracy, intersectionality, and power. The platform offers researchers and practitioners a unique arena to study and foster collective learning and reimagine alternative social-environmental futures.

## Addressing biodiversity loss is a significant governance and social learning challenge

There is compelling evidence of unprecedented and alarming rates of biodiversity loss^1,2^ and the overall trends posing a critical threat for humans at local-to-global scales^3,4^. However, addressing biodiversity loss presents a significant governance challenge. Effective biodiversity governance necessitates collaboration and coordination among involved actors to manage shared resources to meet often-competing needs such as producing food and maintaining habitat for diverse species. Achieving this requires overcoming the absence of direct or immediately observable causal relationships between actions and outcomes. It is a particular challenge to prioritize biodiversity in decision making, especially in light of tangible and short-term benefits from so-called free ride, when one benefits from the resource system without contributing to its longer-term maintenance^5–7^. The Intergovernmental Science-Policy Platform on Biodiversity and Ecosystem Services (IPBES)^1^ identified human activities such as land use change, direct exploitation, and pollution as direct drivers of biodiversity loss. However, observing how, for example, change in land use leads to biodiversity loss is not easy due to delayed and geographically dispersed impacts^4^. Thus, both economic measures (e.g., internalizing externalities—unaccounted costs to other actors and the environment) and regulatory interventions (e.g., developing regulations that create incentives preventing externalities) have practical limitations in addressing biodiversity loss. The latest IPBES assessment^2^ recognizes this and calls for fundamental shifts in views, practices, and structures to address the alarming rates of biodiversity loss and particularly accelerate food systems transformations^8^.

Moreover, because “biodiversity” encompasses the totality and plurality of all living beings, the term entails particularly diverse perceptions, complicating consensus on definitions, issues, and solutions^9^. Under these conditions, social dilemmas, where interests of specific actors clash with interests of the broader system, have a tendency of creating even more room for free ride as preventing, detecting, and compensating for it is extremely difficult. Further, looking at available choices to any individual actor from a historical-institutional perspective^10–12^, it is evident that our actions are bounded by path dependencies (existing norms, infrastructure and policies, networks, etc.) that make it even more difficult to break out of established paths and make new choices. For example, if actors (individuals, communities, societies) have unsustainable consumption patterns with impact on biodiversity in other parts of the globe, breaking path dependencies (existing norms, infrastructure and policies, networks, etc.) and setting up a new governance system that takes into account the costs and either prevents or compensates free ride, especially in a centralized manner and top-down, is indeed a tremendous challenge. Hence, internalization of norms where actors develop their own intrinsic motivation compatible with preventing actions that lead to broader undesirable outcomes^13–15^ becomes particularly relevant for facilitating change in such complex systems.

Games are known as a promising participatory intervention for social and experiential learning about complex social-ecological processes and internalization of norms^16,17^. Well-designed and implemented games can start transformations from within or bottom-up, that is individual actors might adjust their beliefs or develop new beliefs about issues at hand, which then can affect other actions and interactions they are involved in. A number of games that particularly focus on learning about social dilemmas and prevention of free ride have emerged recently, where relatively well-studied social dilemmas (e.g., public goods or common pool resource dilemmas) are framed in specific social-ecological contexts^13,18,19^. Generally, many studies show that context matters when generic games are stylized or framed for a certain social-ecological context, or generally when games focus on facilitating learning situated within specific time, place, situations, and in relation to specific people^20–23^. Likewise, developing a game for facilitating social learning about biodiversity is both a theoretical and practical challenge, as decisions must be made on myriad features of a game that can influence its outcomes both within and beyond the game, ranging from those on game narrative and experience to those on game rules and importance of player attributes^19,22^. Games that effectively facilitate learning about complex subjects, particularly by addressing the multiple layers of underlying social dilemmas to overcome the free ride problem, are relatively rare in the world of experiential games^17,24–26^ and virtually non-existent at the intersection of biodiversity, food, and governance.

## Social dilemmas at the heart of addressing biodiversity loss

Here, we aim to fill this gap by sharing our lessons of developing and piloting Pathbreak: A Biodiversity-Food-Governance Game (www.pathbreak.eu). We present Pathbreak as a proof-of-concept boundary object that operationalizes layered biodiversity–food–governance dilemmas and elicits participant reflection through facilitated debriefing. We focus on the importance of integrating multiple direct and indirect layers of dilemmas for social learning. We reflect on to what extent the new game succeeds in bringing these dilemmas to the attention of the participants using an in-depth analysis of debriefing sessions from five pilots implemented in a three-year Horizon Europe project dedicated to societal dimensions of addressing biodiversity loss. Our specific research questions are: How does Pathbreak operationalize layered biodiversity–food–governance dilemmas? What kinds of first-order and second-order dilemmas emerge in participants’ debriefing reflections? How do participants connect the game experience to real-world biodiversity governance challenges?

A social dilemma is any group situation where one actor is, at least temporarily, better off when this actor does not cooperate while others in the group do; however, if all actors defect, the payoff is substantially lower than if they cooperate^27,28^. As this structure of incentives generates tension between individual and group rationality, such problems were widely analyzed by researchers. The investigations included not only the economic framework of the dilemma^29–31^ but also, among others, the role of monitoring, sanctioning, and institutions^32–35^, communication^36–39^, individual values, mental models, and social norms^40–43^, as well as group identity^44–46^ that moderate the cooperation and competition in such situations.

Research shows that experiential learning through social dilemmas can be particularly beneficial for learning about social processes when facing dilemmas, especially in social-ecological contexts. Bush et al.^24^ highlight that “a degree of firsthand discovery and novelty (e.g., violation of basic assumptions)” when players experience the dilemma situations and the consequences of their decisions may facilitate conceptual understanding of complex phenomena. Bush et al. further note that this is in line with studies on both decision-making in naturalistic settings^47–49^ and educational theory^50,51^. Generally, ecological issues are deeply rooted in social dilemmas, where individuals are inclined or forced to prioritize personal interests over societal consequences^52^. Despite being the core drivers of major social-ecological challenges worldwide^5,6^, this connection often goes unrecognized by many^53^ and also within the latest biodiversity assessments^1,2^. However, understanding this fundamental link between social dilemmas and environmental degradation is essential for fostering effective societal cooperation and shaping public policies that promote human well-being and global sustainability^5,54^. Thus, there is a strong need for enhancing public awareness of social-ecological systems and their inherent dilemmas^55,56^.

In the biodiversity domain the need for social learning games is well documented^57^ especially for ones based on social dilemmas, as well as social-political processes around them. In contextualizing generic social dilemma games (e.g., prisoner dilemma, public goods, common pool resources games), a caution is necessary about designing choices players have to make in the games. The complexity often arises from social interactions and group processes, beyond the underlying formal model of the game that might focus on decisions between sets of limited choices. As Levit and List^20^ put it, “in experiments, the researcher creates a set of rules governing the interactions, chooses the wording of instructions, and defines the set of actions the subject is allowed to take. In stark contrast, in naturally occurring environments, the choice set often is almost limitless and institutions arise endogenously”.

Based on these theoretical considerations and building on what Ostrom^5,6^ described as first-order and second-order social dilemmas, within the domain of biodiversity-food-governance, we identify at least the following three sets of factors that define the key social dilemmas central to addressing biodiversity loss.

### Direct or first-order biodiversity-food dilemma

Although this dilemma is not linear, biodiversity loss is frequently associated with agricultural expansion or intensification, which typically requires more land or intensifies existing land use^58–60^. Biodiversity loss, in turn, negatively impacts productivity of ecosystems, affecting the overall resilience of social-ecological systems—their ability to respond to changing needs and future uncertainties. Increasing numbers of extreme weather events in the last decades are often closely associated with the declining diversity and health of the ecosystems, habitats, and species, which otherwise provide often-vital ecosystem services and perform functions to stabilize climate and to ensure longer-term sustainability of (non-human) nature’s contributions to people^61–63^. At the same time, some literature emphasizes the unavoidable need for high yields in the context of global food shortages due to the detrimental consequences of reduced yields, both for the impoverished communities and countries and in terms of risks associated with further agricultural expansion to meet newly generated import demands^64^. This first-order dilemma presents producers with a choice between lower and higher yields (through expansion, intensification, and their mixtures), while consumers face the questions of food affordability and/or make choices between higher or lower levels of consumption. Each decision must balance the individual and shorter-term payoff with the group and longer-term payoff.

### Indirect or second-order governance and political dilemmas

The governance and political dilemmas focus on how much energy should be invested in communicating, developing new rules, and generally being active beyond one’s own private sphere with an attempt to influence others in a given community. Here, depending on the individual circumstances, the consequences of the dilemma can be substantially different, therefore the term dilemmas is used in plural. It is well known that effective governance in complex social-ecological systems that can address both the equitability and sustainability considerations in the system and ensure participants avoid the worst-case scenarios, requires effective coordination^52^. Individuals in positions of power often invest significant effort to attain their positions and derive privileges from their authority, including pursuing policies that serve public and specific interests. They also endure public scrutiny and bear the risks associated with power misuse or changes in power dynamics, including retaliation. One can also be politically active without getting in a formal position of power, through mobilizing, rallying behind a certain cause or candidate, expressing or withholding endorsement, etc^65^. All of these options also have potential consequences consisting of risks and benefits, including the prospect of being on the losing side, enduring disappointing policies enacted by the supported candidate, being rejected in communities that supported the other candidate(s), and so on. Further, the coordination of providing public goods and maintaining common pool resource systems is often costly in larger scales^66^. When decisions have to be made that change how society prioritizes various objectives—for example, limiting or expanding available land for agriculture, raising or increasing tax for use of public goods or common pool resources, dedicating resources to various issues—one can think of the immediate decision maker, for instance, a governor, or entire society where historical events led to specific norms and values in that society and specific leaders being elected and setting specific political agenda. Considering the state of biodiversity and food as a result of such broader historical social-political developments, it becomes evident that any transformative change in the biodiversity and food domains would necessitate transformative change in the governance domain.

### Place-based intersectionality dimensions

Intersectionality dimensions such as age, gender, education, income, cultural background, religion, disability combined with specific place are known to make opportunities and barriers asymmetric, explain systematically different perceptions of the same phenomenon (e.g., the dilemmas above), and result in fundamentally different individual interactions, sometimes unlike what is predicted by formal theory or models that report mean values or “typical” behavior^67–69^. Thus, contextualization of the game for addressing biodiversity loss might have some general dilemmas, for example of deciding based on individual versus group interest, yet for a very specific person with very specific background in a specific place these dilemmas can mean substantially different trade-offs. For some a public goods game might mean an exercise in economics, while others might be imagining a very specific school that is being constructed in their neighborhood. For some it could mean selecting food over biodiversity for livelihood and avoiding extreme poverty already in times of hardship, for others it could mean selecting food over biodiversity as a matter of maximizing profit and preserving a long-term agricultural family tradition. For some, speaking out might mean an integral element in the battle for power and nurturing democracy, for others it might mean risking their immigration status or attracting personal attacks while already being disadvantaged.

Looking at these dilemmas and factors, it becomes evident that addressing the indirect drivers and underlying causes of biodiversity loss goes well beyond the biodiversity domain. Experiential learning is considered particularly useful for such complex and multiple layers of dilemmas. According to David Kolb, experiential learning is “the process whereby knowledge is created through the transformation of experience. […] Knowledge results from the combination of grasping and transforming experience”^70^. One should note that Kolb uses the term “transforming experience”—that is when experience turns into knowledge through reflection and realization of what was experienced. Kolb^70^ suggests a four-stage experiential learning model where knowledge is continuously created through concrete experience, reflective observation, abstract conceptualization, and active experimentation. In using games for facilitating learning, active testing in the game is often linked to direct experience, reflected upon, and conceptualized in debriefings, which scholars consider as the most important point of learning from the use of games and simulations^71–74^. One can distinguish cognitive (focus on knowing) and non-cognitive learning such as affective (focus on emotional understanding of others and the situation), motivational, relational, normative, sociocultural (focus on understanding or building relationships and trust, norms of interaction, etc.)^19,75,76^. Overall, both David Kolb as the pioneer in experiential learning theory and many who have worked on it over the last decades explain that experiential learning is a transformative process where one readjusts one’s own knowledge and relational system to integrate the new experience.

Thus, transformation in learning for us has two important dimensions—it is a process that effectively transforms the simulated experience into actionable knowledge^70^, and one that in a complex social-environmental context such as in addressing biodiversity loss facilitates learning that goes beyond any single level of social change^2,9^. Our experiences of developing and piloting the Pathbreak game in PLANET4B (see background to the project in the “Methods” section) confirm these multi-faceted societal dimensions of biodiversity on the one hand and games and learning on the other.

## Pathbreak experiential learning platform: Integrating layered dilemmas

Before introducing the Pathbreak framework and how the layered dilemmas are integrated, we should note that the game elements are simultaneously both the result of “translating” the theory of social dilemmas discussed earlier into a tangible and practical framework that allows to experience these social dilemmas and the platform for understanding further insights from experiencing it.

### The concept

**Fig. 1:**
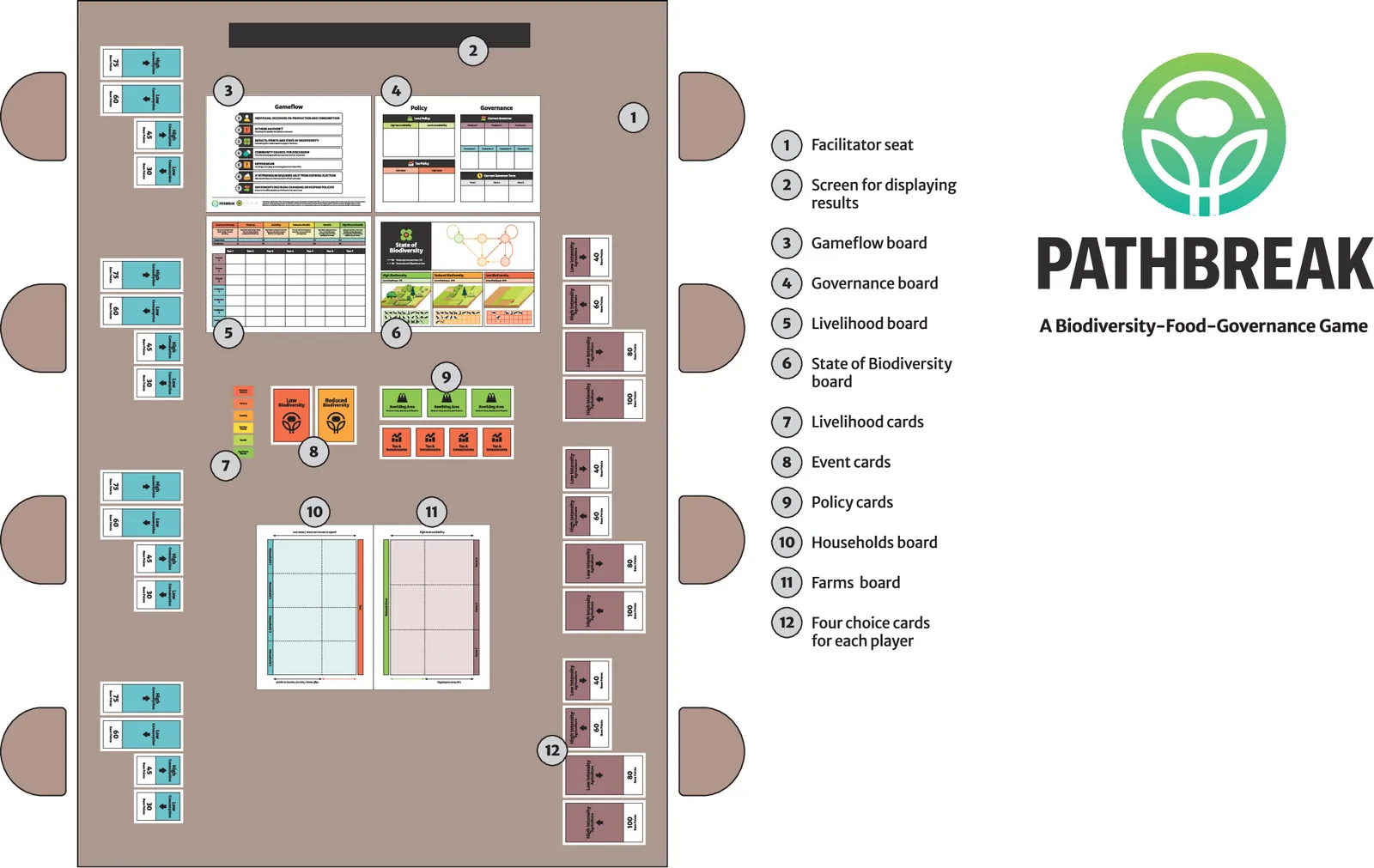
A recommended table layout in Pathbreak: A Biodiversity-Food-Governance Game.

Pathbreak: A Biodiversity-Food-Governance Game immerses participants in a simulated community where they can explore and actively test^70^ the complex interconnections between biodiversity, agricultural practices, and policy-making. The game is called Pathbreak as the participants will face the challenge of breaking the path consisting of unsustainable production and consumption patterns and requiring reconciliation of one’s own, various sub-groups’, and broader system’s interests (resilience of the social-ecological system). The activity runs for approximately 90–120 min, including test rounds, the main gameplay, and a debriefing session. It is facilitated through a combination of physical game boards, role cards, choice cards, and an online Web Application for data input and estimation of points (see Fig. 1, the detailed game materials and facilitator’s manual available at https://doi.org/10.5281/zenodo.17476375).

### The interactive setup and embedding the key dilemma in goal-setting

In the game, one facilitator and seven players—three producers (representing farms) and four consumers (representing households)—sit around a table and make a number of decisions (Fig. 1) (more than one participant can play as one player). At the beginning participants are informed that they can set their own goal for the game. They are also informed that if by the end of the game the state of biodiversity is not in red, one person with the highest points will receive a symbolic reward (e.g., sticker, origami, pen).

### Structured and unstructured decisions

They make decisions that have consequences for their individual livelihood (individual points) and the state of biodiversity. They can communicate and make decisions about governance that can result in policy change (virtually limitless set of decisions except for the limited time, such as to what extent to be politically active and how).

### Selected policies

There are two specific policies in the game. Land Policy applies to producers and can reduce or increase available land for production. The default policy in the first round is high land availability for production. Tax Policy applies to consumers and can reduce or increase net income for spending. The default policy is low tax/high disposable net income.

### Direct first order social dilemma

**Fig. 2:**
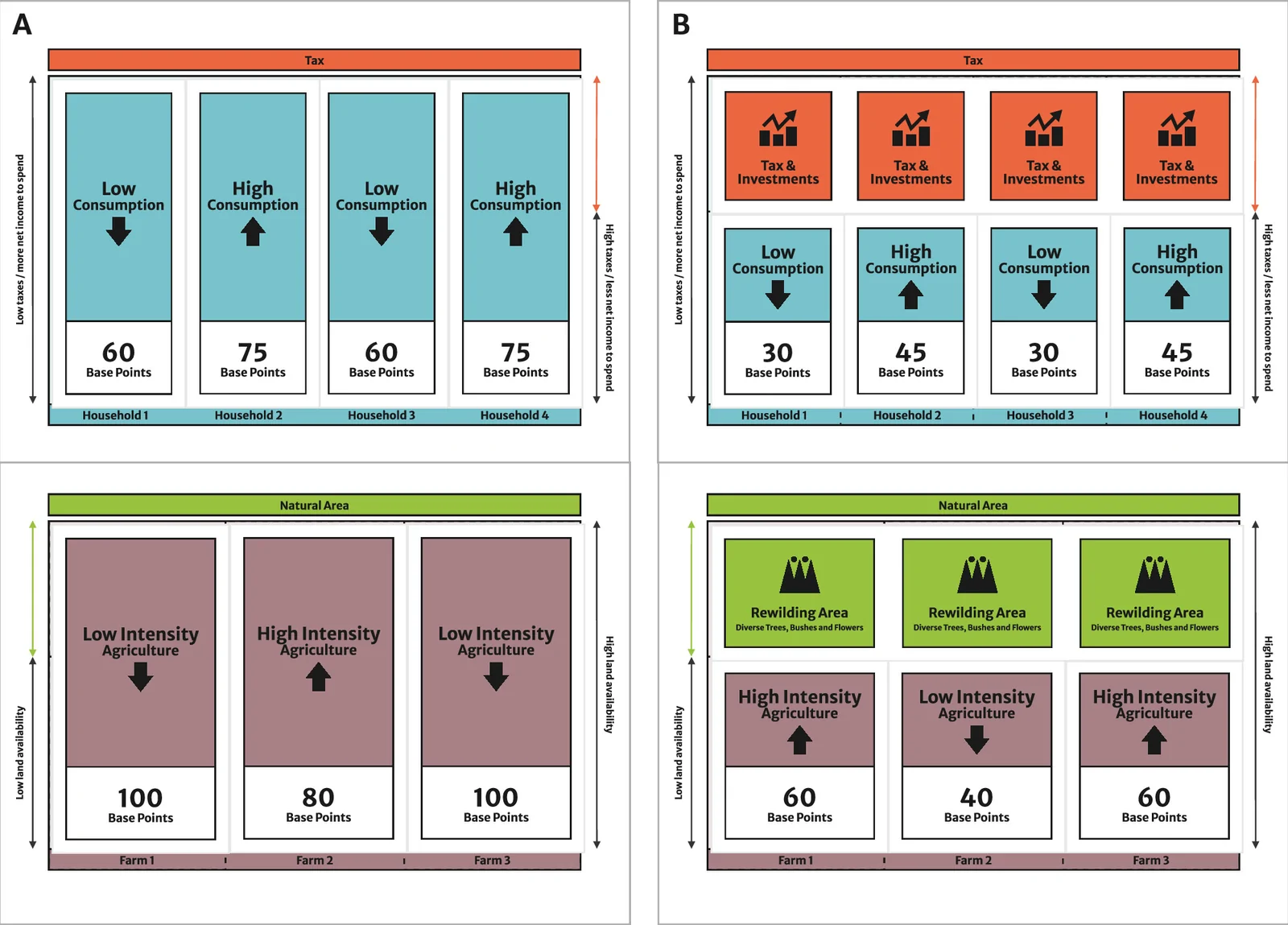
Two examples of what the main game board could look like after placing the cards on the board. **A** Policies are high land availability for producers and low taxes/more net income available to spend for households; and **B** Policies are low land availability for producers and low taxes/more net income available to spend for households. Policy cards are framed as “Tax & Investments” and “Rewilding Area: Diverse Trees, Bushes and Flowers”.

In each round players representing farmers decide on the intensity of production (high or low). Consumers can choose between high and low consumption (Fig. 2). They receive higher or lower individual points accordingly. Points are estimated through a market-based supply and demand model (see “Methods”) that is made available to the participants live via the Web Application.

### Impact on and from biodiversity

**Fig. 3:**
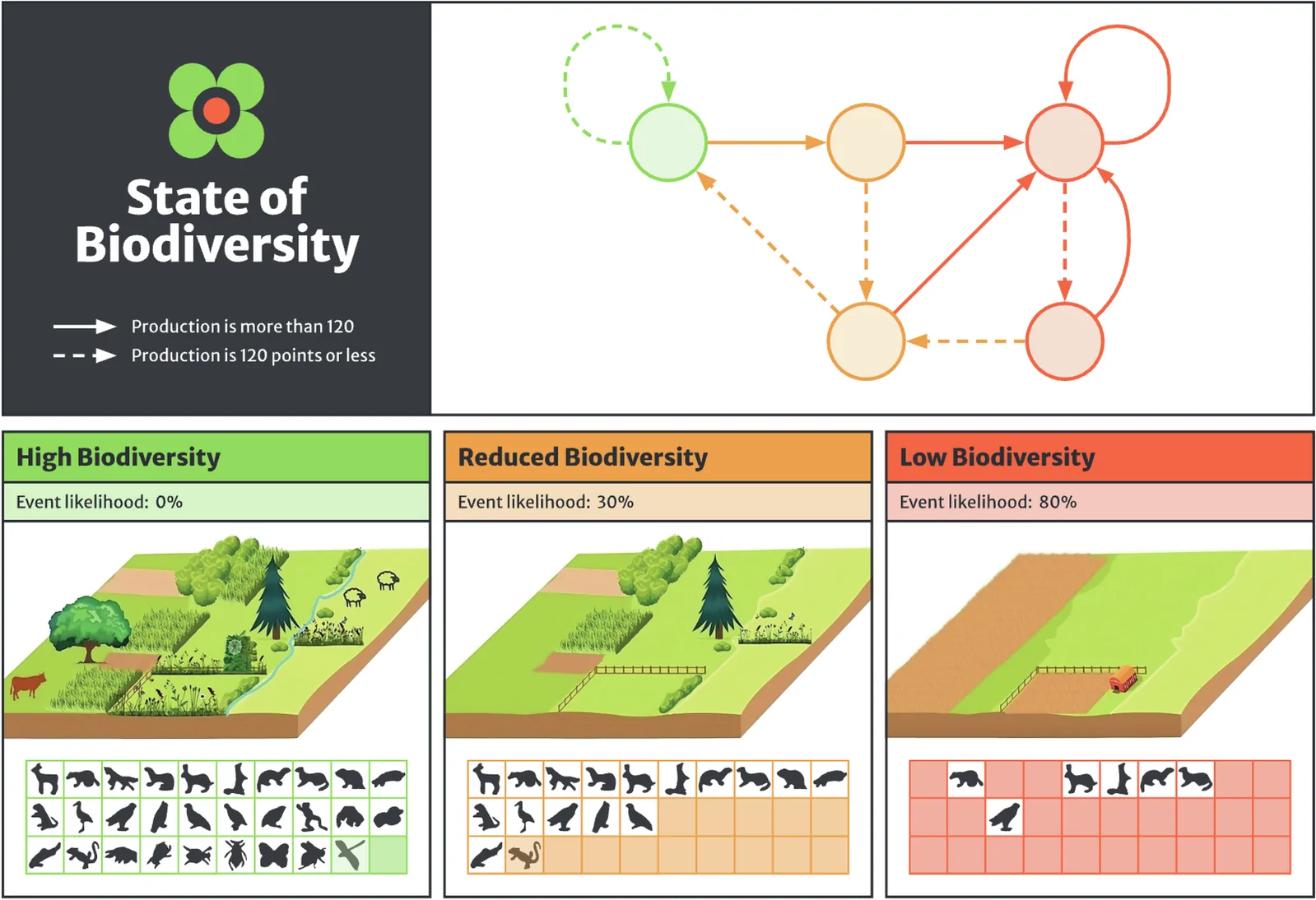
The three states of biodiversity.

There are three states of biodiversity: high (green), reduced (yellow), and low (red) (Fig. 3). Any combination of decisions except one will worsen the state of biodiversity or keep it at low if the players have already reached it. The exception is when the Land Policy is low land availability and all of the three farms produce with low intensity. Two rounds of coordinated action are needed to improve the state of biodiversity and one round of uncoordinated action for worsening it (or keeping it at low/red).

The state of biodiversity has an impact on the likelihood of events that can take place after each round (based on the works revealing the importance of biodiversity for increasing the resistance of ecosystem productivity to climate extremes, see earlier discussion). Events—extreme weather events—penalize all players by deducting substantial points (−80, see the Methods section for calibration details). When the state of biodiversity is high, the likelihood of events is 0%, when reduced 30%, and when low 80%. Players draw from a stack of cards after each round with the color corresponding to the state of biodiversity in that round (yellow or red).

### Indirect second order social dilemmas

Between the rounds, there are community council meetings where players can communicate about anything they would like.

Then players make decisions about the Governor. First, they are asked whether anyone would like to nominate themselves or another player to become the Governor who can then change the policies. Nominees have the opportunity to make a brief statement about why others should vote for them. Once the statements have been made, the players vote for candidates. In case of a tie, if there are more than two candidates, the second round of voting takes place only with the two frontrunners. The frontrunners can make new statements. If there is still a tie, the Governor’s office remains unoccupied and policies remain unchanged.

If there is a Governor, the Governor makes decisions on two policies: Land Policy and Tax Policy—whether to keep or change each of these.

Each elected Governor can serve for the maximum of three terms (three rounds in the game). Once there is a sitting Governor, they can be removed from the office sooner through a referendum. When there is a sitting Governor, the facilitator asks players whether there is a need for a referendum. If the majority votes for referendum, the facilitator asks players to vote for keeping or removing the current Governor before proceeding with the possible new election.

### A structured intersectionality dimension

One of the consumer players is a migrant without citizenship and therefore is not eligible to vote in the referendums and elections, nor to run as a candidate. The facilitator informs players about this during the first round of the game after asking who would like to run for the office of the Governor. However, the facilitator reveals in round five that the migrant’s application for citizenship has just been approved and now this player can participate in referendums and elections with the same rights and responsibilities as the others.

### An unstructured intersectionality dimension

A further (optional) feature is the role cards. After the initial two test rounds and before the actual game begins, each player receives a role card with a short profile. These cards are distributed randomly. They contain information on their more general role (consumer 1, consumer 2, consumer 3, consumer 4, producer 1, producer 2, producer 3) and profile. The profile of each player was generated to make sure that some of the key arguments from real-life social dilemmas (link to direct experience^70^) relevant to the broader topics of the game are available to the players (livelihood and personal background, views towards biodiversity and agriculture, the role of government and policies). However, the facilitator informs that this information is optional and confidential, and it is up to the players to what extent to share and build on the provided profile information. This is done to ensure that the players can still actively bring in their own experiences and thoughts, not necessarily being limited by the pre-defined profile.

### Sanctions to sharpen second-order dilemmas during the game

One further option that becomes available to the Governor later in the game is sanctioning any given one player alongside the decisions on the two policies. If the state of biodiversity is low at the beginning of the round and remains low after players make their decisions in the current round, the Facilitator informs the players that the Governor has emergency powers to sanction someone. If the Governor uses these powers, the sanctioned player loses points (30 points).

### Number of rounds

The game continues for seven rounds (based on the modeled calibration provided in the Methods section). Although it is apparent from the game materials that there will be seven rounds, it is not announced by the Facilitator, to avoid making it salient and nudging players to maximize profit in the last round. This information is neither concealed, and the facilitator can inform about the number of rounds, should the players ask.

### Debriefing and reflections

After the game there is debriefing that focuses on reflections on the game experience, potential real-world parallels, and conceptualization of the experiential learning as a whole - thus completing the Kolb’s^70^ learning cycle.

**Fig. 4:**
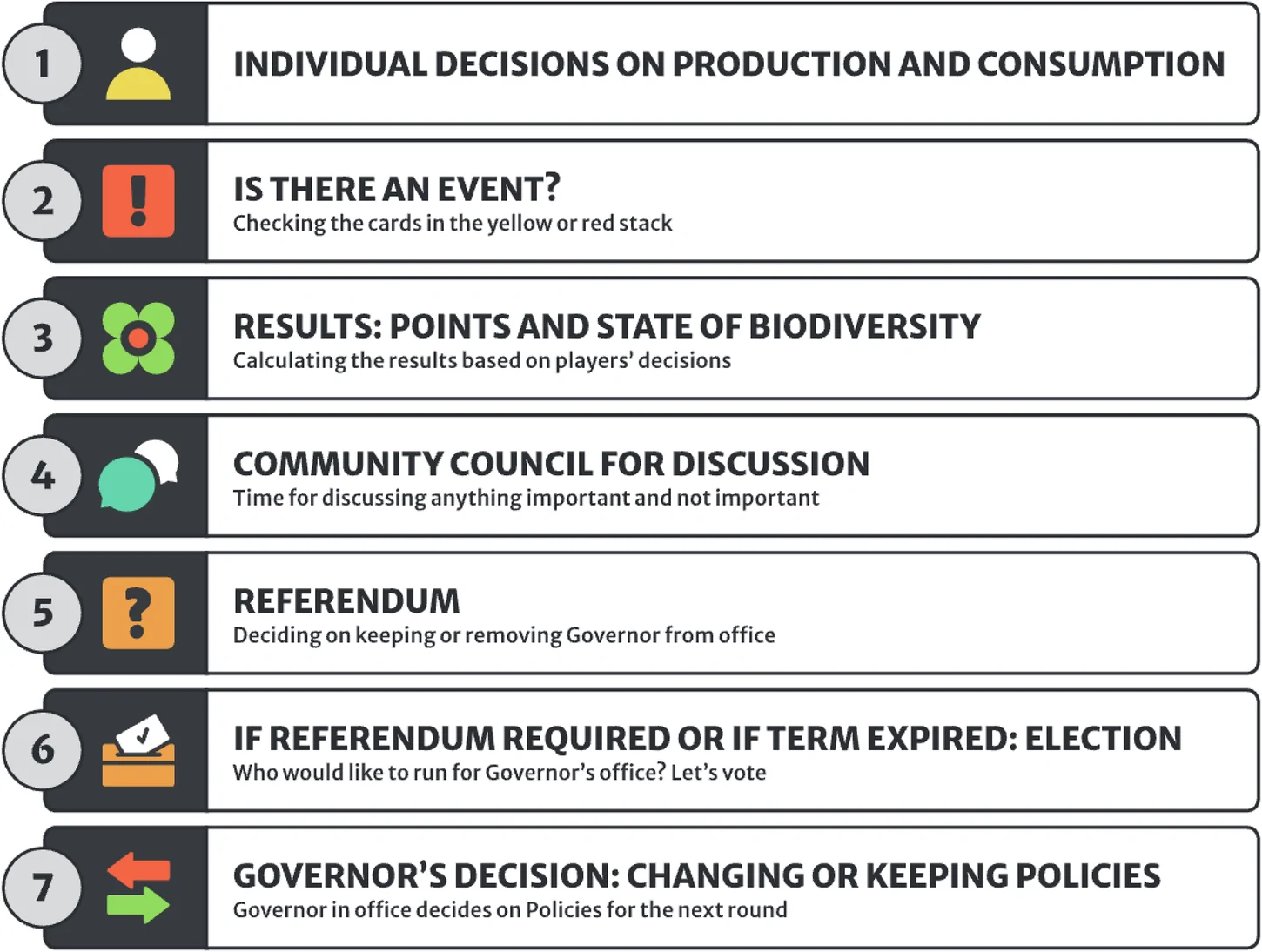
Gameflow for each round in the game.

The overall gameflow for each round is depicted in Fig. 4 and also placed on the table for the players as an orientation.

## Pathbreak and experiential learning cycle

Thus, pedagogically the game is structurally aligned with Kolb’s experiential learning cycle. The gameplay as a whole serves as the *Concrete Experience*, immersing participants in the visceral tension of managing the first order and second order dilemmas. Because the game is played in repeated rounds, it naturally embeds *Active Experimentation* directly into the gameplay loop. Participants continuously test and adapt their strategies through both structured experimentation—such as deploying choice cards, voting, policy decisions, and use of sanctions—and less structured social experimentation, such as adjusting their level of engagement during community council discussions, navigating elections, and deciding whether to actually follow through on stated intentions from previous rounds and potentially face corresponding consequences. Following the gameplay, the debriefing protocol shifts the focus to *Reflective Observation*, providing a dedicated space for players to articulate their feelings (positive, negative, neutral), review their in-game actions, and critically assess the social dilemmas they faced. Finally, the debriefing guides participants toward *Abstract Conceptualization*, allowing them to map their localized gameplay experiences and iterative strategies onto complexities of real-world biodiversity governance and broader theoretical thoughts on how society can or should address challenges such as biodiversity loss.

## Real-world complexity and simplification in Pathbreak

The game is intentionally designed around a core dilemma: the trade-off between short-term gains from high production and consumption and long-term sustainability linked to biodiversity. To make this dilemma salient and experientially accessible, several aspects of real-world systems are deliberately simplified. First, ecological dynamics are represented through an aggregated biodiversity indicator, rather than multiple species, habitats, or ecosystem services. Second, players are given a limited set of policy and decision options, each with relatively strong and transparent effects on system outcomes. Third, actor roles and socio-economic diversity are simplified, focusing on a small number of asymmetric roles rather than a full range of stakeholders and institutions. By reducing the number of variables and decision pathways, the game provides clear and immediate feedback, thereby shortening the experiential learning loop (in the sense of Kolb). Within the limited number of rounds, participants can observe how repeated choices lead to system degradation, attempt to reverse these trends through cooperation, and experience how such recovery efforts may be undermined by individual incentives, role asymmetries, and probabilistic disasters.

Despite its abstraction, the game is designed to reflect several well-established real-world dynamics in biodiversity–food–governance systems. At its core, the model captures a “business-as-usual” trajectory (e.g., refs. ^1,2^), in which increasing production and consumption generate short-term benefits while progressively eroding biodiversity. This reflects widely documented trade-offs in agricultural intensification. The game operationalizes this through a feedback structure in which declining biodiversity increases the probability of adverse events, representing real-world risks such as ecosystem service loss, climate-related shocks, or system instability. The game also embeds multiple layers of social dilemmas. Sustainable outcomes require coordinated efforts across actors, as unilateral actions (e.g., one farmer reducing production) are insufficient to shift the system trajectory. This mirrors real-world challenges of environmental governance, where individual short-term incentives often conflict with collective long-term benefits. Additionally, the model includes elements of social dynamics, such as asymmetries in roles and influence over decision-making processes, as well as the possibility that individual “defection” can undermine both ecological outcomes and social trust. Together, these mechanisms correspond to key real-world phenomena, including externalities in agricultural systems, tragedy-of-the-commons dynamics (collapse in the absence of communication and coordinated collective action), delayed ecological feedback, and the overall importance of governance structures in managing coupled human–natural systems.

We should note that pathways such as agroecology, regenerative agriculture, or biodiversity-compatible intensification are not explicitly modeled in the current game design. This is an intentional design choice rather than an omission. Incorporating these approaches would require representing multiple alternative production systems, heterogeneous landscapes, and more complex policy instruments, which would substantially increase the number of rules, decision points, and possible system trajectories. Such an expansion would risk obscuring the core learning objective and reducing the clarity of feedback within the limited time frame of the activity. The current design prioritizes portability, ease of facilitation, and the ability to complete multiple rounds, allowing participants to observe system dynamics and potentially shift trajectories within a single session. Importantly, the absence of these pathways in the formal model does not mean they are excluded from the learning process. On the contrary, the game is designed to create a conceptual space in which the need for such approaches becomes apparent. During the debriefing phase, participants are encouraged to reflect on the model’s simplifications. In this way, the game functions as a foundational abstraction and a boundary object, while the broader activity integrates more complex and context-specific knowledge and experience, shared by the participants themselves.

## Proof of concept: results from piloting and debriefing

### Experiencing the direct biodiversity-food dilemma in Pathbreak

**Fig. 5:**
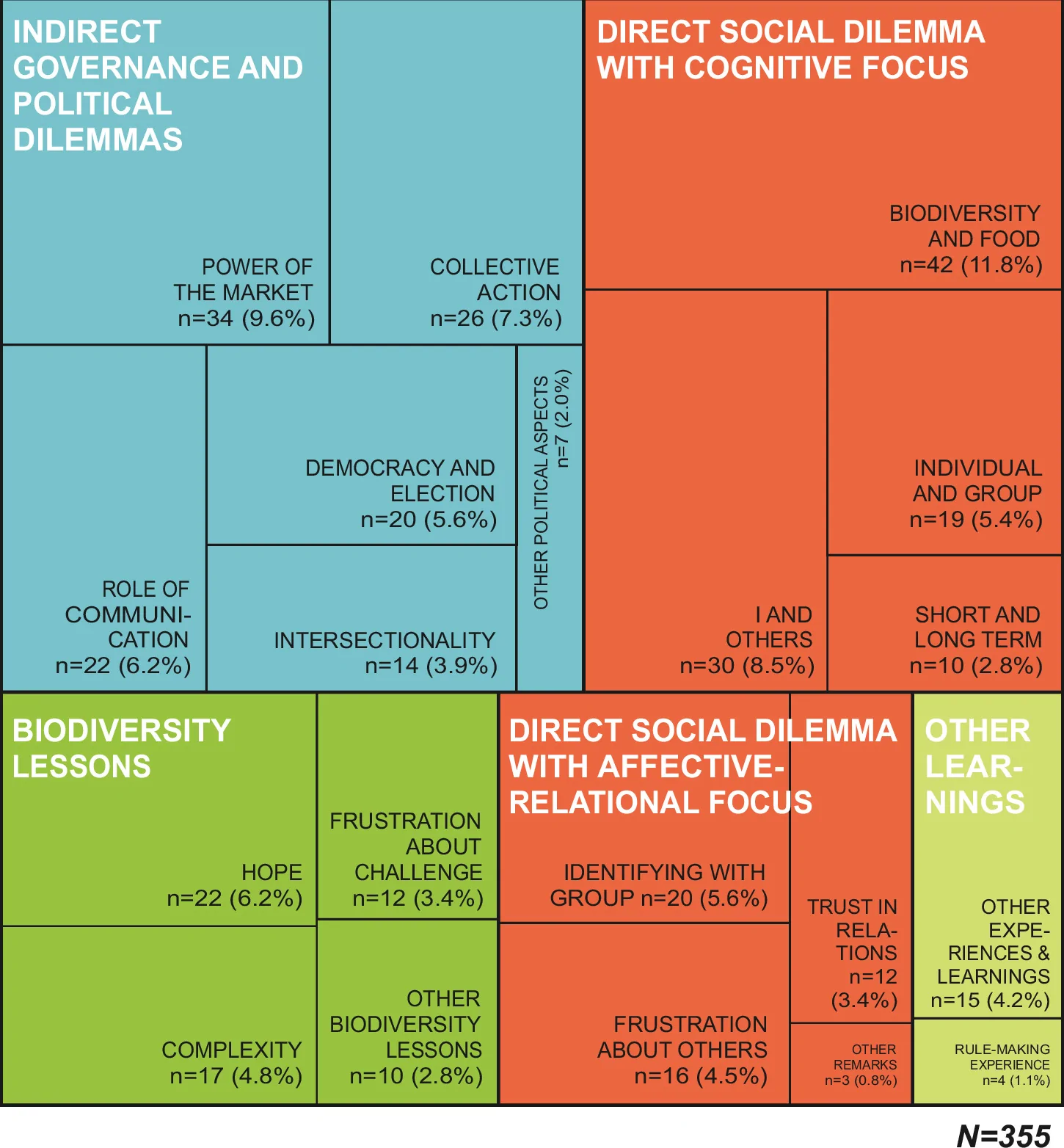
Frequency and distribution of themes emerging during the post-game debriefings.

Debriefing analyses demonstrate that the game’s architecture operationalizes direct social dilemmas as intended. Participants consistently identified the structural friction between individual profit-seeking (via intensified production or consumption) and the collective objective of sustaining biodiversity to mitigate extreme weather events. The complex relationship between biodiversity and food was the most frequently discussed theme in the debriefings (Fig. 5). It seemed also clear that participants experienced several layers of individual versus group dilemma—in the sense that there were nominally two groups of producers and consumers initially, but during the game players would realize that there were often points of clash and cooperation also within various sub-groups. They reflected on the dilemmas in personal terms, that is “I and others”, as well as highlighting broader clashes in “individual and group” or “short-term and long-term” interests.

The way participants described their experiences also allows us to distinguish roughly between two types of social learning: cognitive and non-cognitive. Cognitive learning about the dilemma manifested itself as participants tried to make sense of the dilemma, for example by trying to calculate or figure out how the economic model behind the game would predetermine the game outcomes. Non-cognitive learning about the dilemma manifested itself in the way the participants expressed their frustrations by emphasizing the feelings, emotions, the role of trust or lack of it, and how they felt they could or could not identify with the group or the others, highlighting the behavioral dimensions of economic decision-making^77^.

There were also a number of valuable critical perspectives voiced by the participants. One of the key critical points was related to how the game’s design forces players to choose between profit (individual points) and biodiversity, at least implicitly neglecting the possibility of achieving both, which might in turn reinforce the image of the current unsustainable consumption and production patterns. This is an important critique for at least two reasons. First, it is true, as noted earlier, there are non-linear relationships between biodiversity and food, and these should not be neglected. However, that this discussion emerged serves the purpose of Pathbreak—to experience prevailing unsustainable paradigms and talk about possible ways out. Second, the participant’s perception that the game forces players to choose between profit and biodiversity is rather a disappointing news for the developers as participants in the game face myriad invisible choices well beyond biodiversity and food: to be active or not, to communicate or not and to what extent, to run for the political office or not, to enact various policies or not, to support specific candidates or not, to bring specific issues to the attention of the community council or not—the list goes on. Of course, the fact that the participant did not notice that there were also these non-biodiversity and non-formal choices at all times might signal the game’s weakness in facilitating learning. Although, one could argue it is a good simulation of contextual reality where actors might not realize the presence of these rather invisible higher-level political-institutional choices^11^ (see more on this next). Further, while at least one participant perceived the game extraordinarily difficult, particularly in terms of how difficult it was to recover from extreme weather events, another participant found the game limited in its parameters. However, both of these observations are in line with the intended design of the game.

### Uncovering the indirect governance and political dilemmas in Pathbreak

The pilots and debriefings showed that the participants questioned dilemmas they encountered beyond biodiversity and food. They discussed the role of communication, building relationships, intersectionality—privilege or lack of it originating from one’s background, power of the market, difference between being a consumer and an active citizen, and the value and limitations of elections and democracy. They also talked about the complexity of how policies are made, and some examples of how changes made by a new government, even if beneficial in some sense, might not be immediately apparent due to the time required for implementation and the legacy of previous administrations. Hence, they also discussed how political campaigns did not necessarily focus on what could be actually delivered. One participant formulated it: “…at the end of the day, it was more about agreeing between the people. I mean, it showed that that improvement comes from agreement [process], in between the people, among the people, that power” (Debriefing 3, lines 1672–1674, p. 48).

**Fig. 6:**
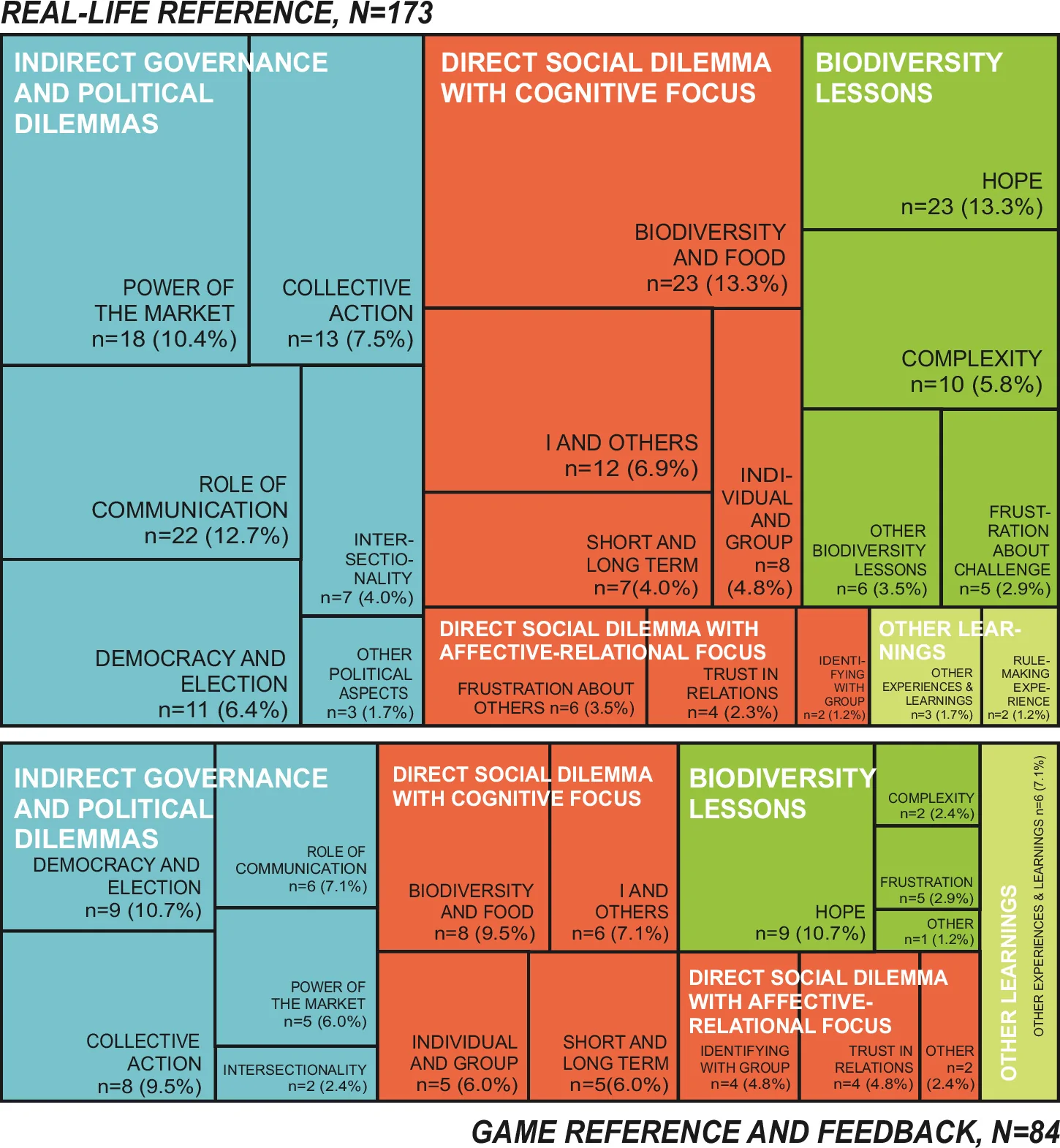
Frequency and distribution of themes by references to real-life and game mechanics.

While reflecting on these, often-underestimated, rather abstract elements of the common-pool resource games, the debriefings also showed that the participants made real-life references across all themes (Fig. 6).

The participants highlighted the game’s potential for improving policy-making. This would be in line with the literature that highlights the value of games for facilitating stakeholder engagement^78^. One participant stated: *“I think it would be really useful for people making local land management decisions. I think where, so I live in an area of sort of lots of high conservation interest, there’s a lot of conflict between the local community groups, councils and people like the National Trust and NGOs and big landowners. And I think when they’re having their big decision making, two-day workshops, it might be nice to play that game right at the beginning. That’s a sort of war game thing. And then they go into their negotiations maybe afterwards”.* (Debriefing 1, lines 530–536, p. 16).

There were also those who were connecting their game experience with real life, while, to some extent ironically, being frustrated that the game was not realistic enough: *“There were some elements that, uh, frustrates me, which I struggle to connect with, let’s say, real life. Other than the fun, and joy that comes with it. There’s no question of this. But, for example, I question myself, why do we start from green? Uh, because it’s frustrating that actually in real life we’re not, realistically speaking, in a green area, right? It feels like we are in a red area somewhere. Um, and of course it does, gives the, all the way back, gives the impression of a long road ahead to reach the green space, so to speak. But still, the frustration comes when we’re not there. We’re told, all the way, red and so on. So, these elements give me some frustration to connect with real life. Not, again, not touching upon the fun part and then everything else that comes with it, which is also great”*. (Debriefing 1, lines 33–41, p. 3).

### Intersectionality and identity in governance

A defining feature of the Pathbreak simulation is the deliberate introduction of power asymmetries through role cards. In particular, the inclusion of a consumer role representing a migrant without voting rights served as a primary driver for discussions regarding political legitimacy and environmental justice. In several pilot sessions, this role acted as a “governance bottleneck”, where the excluded player could influence community dialog but remained legally disenfranchised during formal referendums and elections.

Participants reacted to this legal barrier with a mix of frustration and strategic adaptation. The eventual granting of citizenship and voting rights in the fifth round often functioned as a critical turning point, shifting the community’s focus from mere economic survival to the broader ethics of democratic participation in environmental decision-making. This transition illustrated that addressing biodiversity loss is not solely a matter of scientific or economic consensus, but is deeply intertwined with the inclusion of marginalized voices and the dismantling of institutional barriers.

We have also observed that participants often started the game making references to the profile information and gradually merged it with the knowledge from their own backgrounds. In addition, while the sample of 50 participants across five pilot studies is informative for a proof-of-concept, the heterogeneity of the sample (e.g., experts, students, community members) raises important questions. Differences in prior knowledge, and “cultural capital” may have influenced participants’ engagement with the game and the resulting learning outcomes. One should be aware of the self-selection bias - all participants were already either open for experimenting with such topics and activities or embedded in a professional or broader social environment where trying a game like this was not something completely out of ordinary. It would be useful to test the game with audiences with little familiarity with the topic and across cultural contexts. Yet, the fact that all games revealed the multiple layers of the embedded dilemmas, despite the heterogeneity, gives a good indication that these are genuine and appropriate dilemmas of biodiversity-food-governance for consideration in multi-actor settings with diverse backgrounds.

### Lessons specific to tackling biodiversity loss

Based on the analysis of the debriefings, the overall impression in terms of lessons specific to tackling biodiversity was related to the problem’s complexity and frustration coming from the realization of this complexity, which is in line with the literature that highlights games being particularly useful for facilitating learning about complex subjects^79^.

*“I think for me […] I use that word frightening. […] it made me think about the bigger picture and […] again, not so much for me, but […] about the next generation […] and how or what we can potentially do to influence things going forward. So, if nothing else, the game certainly made me think more about what I can do going forward and maybe a desire to do more quickly”*. (Debriefing 1, lines: 695–700, p. 21).

Some participants shared thoughts that indicated they were still hopeful, despite the complexity and frustration, what seemed to be rather an endorsement for the game and relatively deep discussion they had just experienced.

*“Yeah. So, then I think it creates […], kind of sets the ground for more ideas, like just a brainstorming session and discussion session. Just information sharing, especially with the […] group. That would be nice to get kids from different places to exchange on a topic that’s common to all of us. So that’s really nice, I think, about this game”*. (Debriefing 1, lines 744–747, p. 21).

Looking at these two illustrative quotes, one can also note that the participants thought the game could be useful for different age groups in terms of both making one think about future generations and helping younger generations to learn about layered dimensions of the topic.

### Learning across contexts with different emphasis

**Fig. 7:**
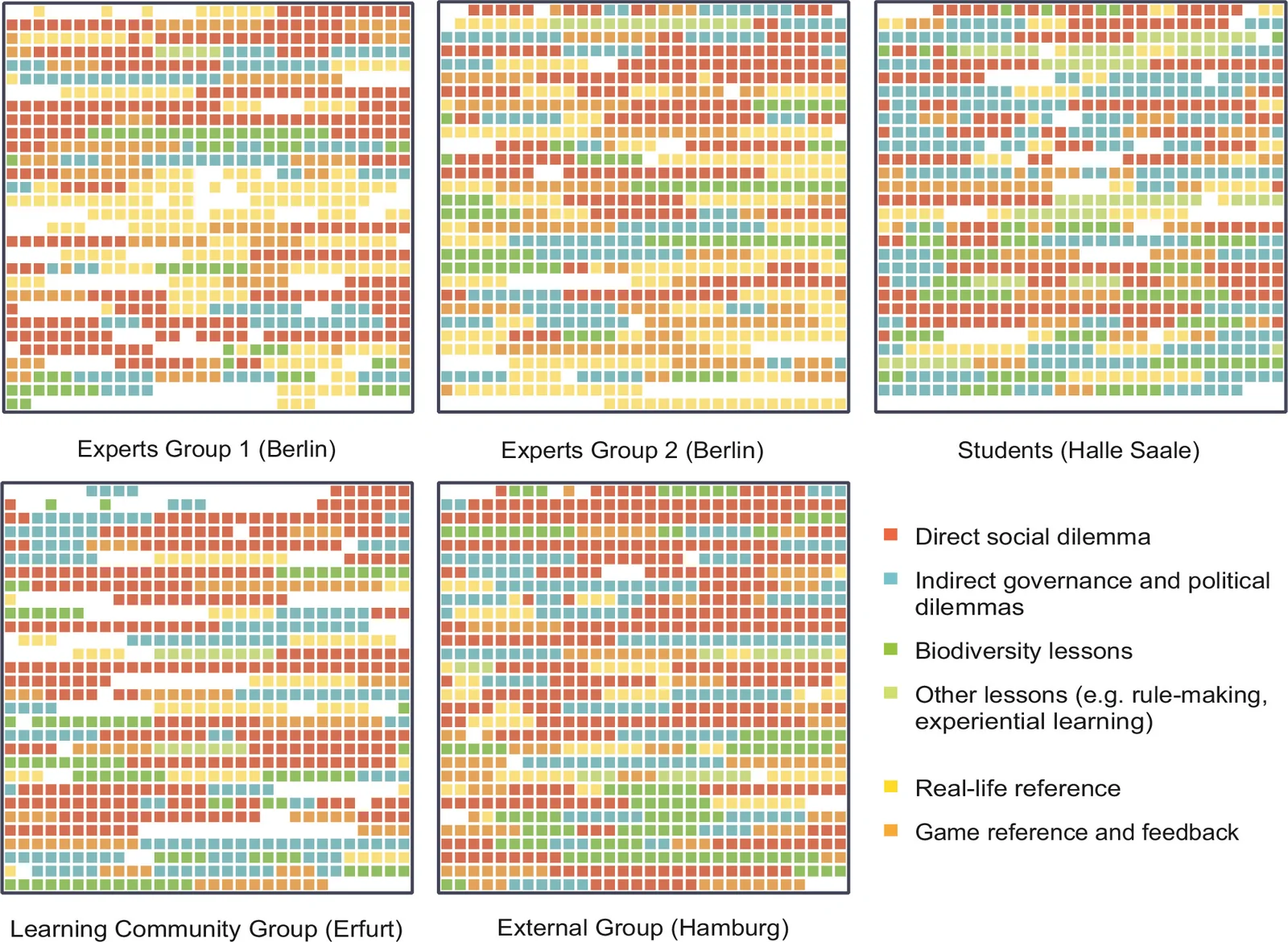
Debriefing focus diagrams for five pilot studies illustrating thematic composition and dynamics. Each figure represents the changing themes over time, where the start of the debriefing is at the top left and the end is at the bottom right.

We have also witnessed some similarities and differences across the debriefings. Figure 7 shows dominance of one or another group of themes. The game and the debriefing generally led to a frame that consists of social dilemmas, political processes, complexity of biodiversity. The figure also shows that it still allows participants to develop their emphasis which can vary considerably. While experts tend to talk about the game as a tool and provide more feedback on its design, students talk more about politics and governance, social dilemmas, and specific biodiversity lessons, which is in line with their generally active learning phase of life. The learning community members, some of whom played the game twice, talked more about the key social dilemma. The external group focused on the key social dilemma most, then politics and governance of it, and then lessons specific to tackling biodiversity loss. It should also be noted here that while the transcripts from the debriefings with the experts were more challenging to code as they seem to be in constant search of topics at higher level and therefore not necessarily address themes directly, the students’ group was easiest to code and analyze. We also see that some of the discussions were very detailed and free-flowing, while others were shorter and more focused, once again indicating that the design of the game had both the structure to talk about specific dilemmas and flexibility to bring in the perspectives of the participants.

## Discussion

Addressing biodiversity loss requires grappling with complex layers of social dilemmas that link individual action, collective outcomes, and institutional arrangements. Pathbreak: A Biodiversity–Food–Governance Game addresses this challenge by providing an experiential setting where participants confront the tensions between short-term individual gains and long-term collective well-being. We acknowledge that the presented work has been piloted in a small number of cases, yet we argue that it serves as a proof of concept, and its value lies in the *framework* and the *qualitative depth* of the boundary object, rather than statistical generalizability. In line with Nobel Laureate Elinor Ostrom’s seminal works on governing the commons, our findings show that participants frequently experienced and debated both first-order dilemmas (production/consumption vs. biodiversity) and second-order dilemmas (governance, trust, and coordination). These multilayered dilemmas are rarely addressed simultaneously in biodiversity education and learning tools, yet they lie at the heart of biodiversity decline^1,2^.

A central lesson from the pilots is that players did not merely interpret the game in ecological or economic terms, but also engaged with broader political and normative questions: power asymmetries, governance legitimacy, and the difficulty of sustaining collective agreements. This echoes Ostrom’s insight that overcoming social dilemmas depends on collectively crafted rules, communication, and the internalization of reciprocity and trust^5^. Several participants highlighted that “improvement comes from agreement between the people”, resonating with Ostrom’s emphasis on social capital as a condition for cooperation capable of overcoming dilemmas. That the game sparked similar reflections across diverse groups—from students to practitioners and experts—suggests that it has a broad potential to support social learning and dialog on biodiversity-food-governance dilemmas.

At the same time, our pilots highlighted the importance of being aware of critical design limitations when using experiential games: some simplification or abstraction is necessary, but one should avoid oversimplification. Our observations and experiences show that the simplified ecological dynamics and the associated hysteresis effect embedded in the game, and as observed in many ecosystems^80^, have triggered reactions similar to what one would expect in the real world. Systems that pass a tipping point do not necessarily recover when pressure is released. That takes additional effort and/or time. Our experiences in the pilots are in line with the previous collective action games with tipping points^21,81–90^. However, most other studies do not include events happening dependent on the state of the ecosystem (but see Janssen et al.^66^), and Pathbreak extends the current literature by offering a social-dilemmas game on biodiversity with a dynamic ecological feedback mechanism. The ecological dynamics in the game is simple compared to real ecosystems also because the goal is not to create a model with similar complexity as reality. As we have seen in our pilots as well, some participants might find it challenging to understand the dynamic processes even within this simplified environment. For participants, these simple dynamics are typically a challenge to comprehend within a short amount of time. In fact, research has shown that humans, including graduate students at MIT, have difficulty managing dynamic systems^91,92^, and some scholars have proposed that the tragedy of the commons might be caused by people’s inability to understand dynamical system instead of selfish behavior^93^. To avoid that we measure confusion instead of intended decisions, a simplified ecological model that captures relevant dynamics for our purposes is therefore a necessity. This is still without adding the dynamics stemming from the multiple layers of social dilemmas. The pilots demonstrate that the game functions as a sandbox for social interaction which enriches the existing simplicity in the ecological system with social complexity. In contrast to other studies where all players have the same role, this study demonstrates how integrating asymmetric roles, power imbalances, economic inequality, and intersectionality dimensions such as migration in a stylized form can generate virtually limitless scenarios of cooperation and conflict that can fundamentally re-shape how players address the challenges of the underlying dynamic ecological model.

Finally, our pilots show the importance of seeing the game not as an end in itself but as a part of a broader activity. Its primary purpose should be to provide a shared experience that informs subsequent discussion and debriefing. In line with Kolb’s experiential learning cycle, our pilots confirm that the debriefing phase enables participants to connect the model to real-world contexts, reflect on its limitations, and engage in learning and exploration of often-limitless scenarios. This stresses the importance of careful debriefing where perceived limitations can be critically discussed in depth. We should further note, given that the game developers also facilitated the sessions and conducted the primary analysis, recognizing and mitigating insider bias was a methodological priority for this study. The developers’ deep familiarity with the game’s mechanics was essential for accurately mapping participant actions to the intended social dilemmas and keeping in mind that the game experience should be addressed in the subsequent debriefing. Several additional safeguards were implemented to prevent this positionality from unduly influencing the results. During data collection, facilitators minimized their interference by adhering to standardized scripts for the introduction and rules explanation, maintaining a pre-defined and observational role during actual gameplay. To counter the inherent power dynamic during the subsequent debriefings, facilitators explicitly encouraged feedback, including critical concerns and recommendations for future improvements, empowering participants as expert evaluators to disrupt the tendency for polite, confirmatory responses. Furthermore, to ensure analytical distance, a triangulation strategy was employed during the coding process: the coding team always included a researcher who was not part of the pilot but acted as “a critical friend” during weekly meetings. This way we ensured that the primary coder’s interpretations are actively challenged, and that the final themes—especially regarding deviant cases where participants challenged the game’s premise—remained strictly grounded in the empirical data rather than the developers’ original design intentions. Thus, future iterations could explore game experience with facilitators not involved in game development. Similarly, one could imagine exploring more nuanced pathways where biodiversity-compatible production strategies are viable, but it would be important to preserve the clarity of the dilemma structure. Likewise, it should be acknowledged here that the presented study captures immediate post-game reactions. Further research is needed to generate evidence regarding the long-term retention of learning or its translation into real-world change in views and actions.

Importantly, our findings from the pilots point to the contextual nature of learning. As path dependence and bounded rationality literature notes^11,12^, actors interpret dilemmas through the lens of their histories, institutions, and identities. In Pathbreak, participants drew on personal experiences of land conflicts, consumer choices, or political activism, illustrating that the same formal dilemma can carry different meanings across settings. This underscores once again the need to treat Pathbreak not as a “model of reality”, but as a boundary object—a tool for eliciting situated knowledge and facilitating dialog in multi-actor settings.

By integrating direct biodiversity–food dilemmas with indirect governance and intersectionality dimensions, Pathbreak extends the repertoire of social dilemma games beyond classic public goods or common-pool resource framings. The design advances the agenda set by Elinor Ostrom and others to move beyond stylized economic models and to embrace the complexity of real-world governance of shared resources, environment, and place.

Taken together, the pilots reveal that Pathbreak shows promise in fostering both cognitive and affective learning, prompting reflection on trade-offs, power, and pathways for collective action. Once again, while the present study is limited to five pilots, it builds on decades of expertise from academia, policy, and practice. The in-depth analysis demonstrates the game’s potential to contribute to biodiversity-food-governance debates by making abstract dilemmas tangible. Future research could test the game in decision-making contexts with practitioners and policymakers with wide-range of backgrounds, and explore evaluation of learning outcomes across cultural contexts and social groups.

To move from experiential learning to systemic impact, we propose a “Theory of Change” where Pathbreak can potentially serve as a catalyst for better informed policy negotiation. Our findings suggest the game could be beneficial not only for students and experts studying social-ecological challenges, but also as a precursor to formal stakeholder workshops as it creates points of references to key social dilemmas behind addressing a complex social-ecological challenge. By providing a low-risk environment to test the social legitimacy of interventions and multiple scenarios in repeated rounds, Pathbreak could offer a practical tool for stakeholders tasked with developing and implementing international goals, such as the targets set in the Kunming-Montreal Global Biodiversity Framework or to be developed for post-2030 Sustainable Development Goals. The game’s ability to expose “invisible” institutional strengths and barriers makes it potentially applicable for navigating the localized land-use conflicts inherent in scaling up protected and rewilded areas. By immersing actors in the layered dilemmas of trust and reciprocity before negotiations begin, Pathbreak could potentially de-escalate “us-versus-them” mentalities common in land-use conflicts. Specifically, beyond research and education, we recommend testing the tool further by integrating it into:

- Local land and environmental management decisions: Facilitating dialog between community groups, municipal councils, and NGOs to reveal the “invisible” political-institutional choices that shape land use and environmental governance.
- Transdisciplinary science communication: Bridging the gap between STEM-focused biodiversity data and the social-political realities of implementation.
- Policy simulation laboratories: Using the game as a low-risk environment for policymakers to test the social legitimacy of new regulations, such as land-use taxes or rewilding mandates, before their real-world rollout.

Overall, Pathbreak illustrates the promise of social dilemma games as laboratories for experiencing practically limitless scenarios of how one can address social-environmental challenges and reimagining what is possible. It operationalizes Ostrom’s insight that people can achieve outcomes “better than rational” when reciprocity, trust, and dialog are fostered^5^. This is particularly fitting as Ostrom and her colleagues’ advances in institutional scholarship served as the foundation for the landmark IPBES global assessment^1^ that stressed the importance of addressing indirect drivers of biodiversity loss. More broadly, embedding such tools within broader participatory processes—involving citizens, scientists, and policymakers—can be illuminating for assessing their broader contextualized impacts^94^. It may likewise help bridge the persistent gap between biodiversity/STEM science and the societal transformations urgently needed to halt and reverse biodiversity loss while democratizing the science itself and making it more equitable^95,96^.

## Methods

### Background to the project

The Project PLANET4B, funded by the European Union’s Horizon Europe research and innovation programme, grant agreement No 101082212 (2022-2025), as detailed on www.planet4b.eu, was an initiative focused on understanding and facilitating social change to foster improved biodiversity prioritization in decision-making at individual, community, and institutional levels (see also Soliev et al.^9^). PLANET4B is an acronym that stands for understanding Plural values, intersectionality, Leverage points, Attitudes, Norms, behavior and social lEarning in Transformation for Biodiversity decision making. By integrating interdisciplinary research methods, the project aimed to identify key institutional and behavioral drivers and develop innovative strategies that promote responsible actions across various sectors and better take into account potential social and environmental externalities. As a transdisciplinary project, PLANET4B also collaborated with diverse stakeholders, including policymakers, businesses, and communities, to analyze and create practical solutions that support processes towards a more biodiversity-rich and socially equitable future. Experiential learning has been one of many closely analyzed, explored, adopted, developed and piloted methods in PLANET4B.

### Game calibration: theory, the goal, and the context

In Pathbreak, players face the stylized version of the direct social dilemma. We designed it in a way that short-term profit maximization leads to high production and consumption decisions. In the long term, if producers follow this path, it leads to negative feedback from the environment—a higher susceptibility to extreme weather events. They provide penalties for the community when biodiversity is reduced or low. To emphasize the dilemma, we decided to set a sustainable level of production at the lowest production decision.

From the perspective of a single round, the value for the production is matched with the consumption. In theory, an imbalance between the two can create a shortage or surplus that will benefit either producers or consumers through the increase or decrease of the value of one unit of food in the market. Still, it affects the group payoff for the specific type of players. Within the group, the payoffs are distributed proportionally, according to the decision of each player. It means higher individual production/consumption, when all other elements are kept stable, leads to a higher individual outcome. Therefore, each player has an incentive to increase their production or consumption. If all decisions are maximized no player has an incentive to change their action, thus creating Nash equilibrium. This way, rational players should not create artificial shortages or surpluses. Finally, from the perspective of a single round, Nash equilibrium is not affected by the occurrence of an event. Even if there is a probability of a penalty, it does not depend on the actions in the current round. Therefore, maximizing production or consumption increases the expected payoff even in the face of potential events that can reduce payoff (extreme weather events).

**Fig. 8:**
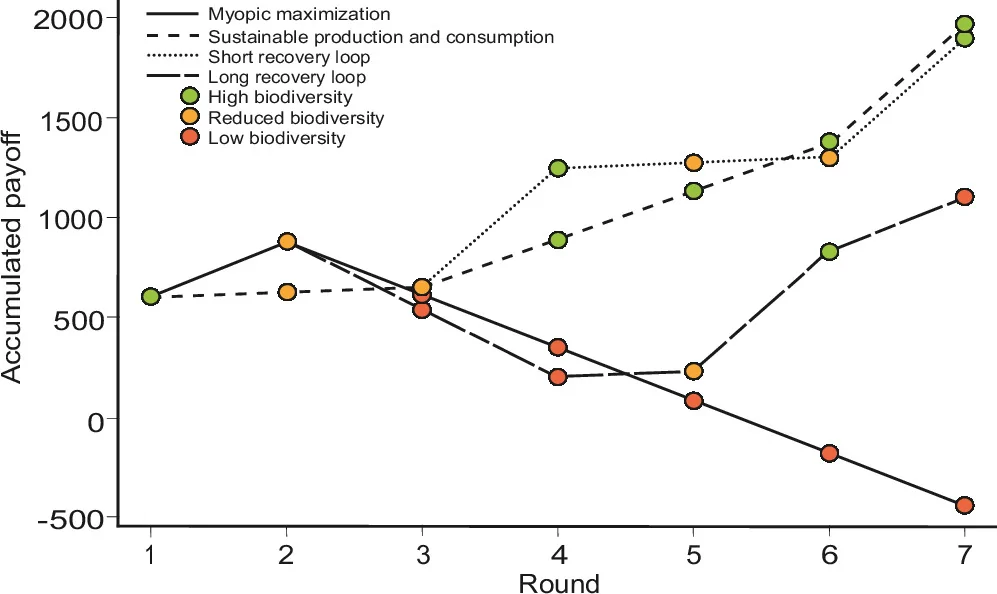
Calibration of accumulated payoff and biodiversity for benchmark strategies.

To calibrate long-term solutions, we used a set of four strategies as a benchmark (Fig. 8). The first two are derived from the general idea of social dilemma. Myopic maximization means that all players follow the single-round Nash equilibrium, and the policies are set for maximum production/consumption. On the other hand, a sustainable strategy means that all players choose in unison sustainable production and consumption along with the matching policies. Alongside these two strategies, we added two more, to have a better understanding of the potential results. The first one shows a short recovery loop, where players maximize their production/consumption when biodiversity is high and switch to sustainable production/consumption when it is reduced, allowing it to recover. The second one follows a long recovery loop, where players maximize their actions until the biodiversity is low. Then, they switch to sustainable production/consumption until it recovers to a high level. At this point, they start maximizing their actions again. Therefore, players create a long cycle of maximizing production and recovery from the low biodiversity.

After establishing these benchmark strategies, we were able to adjust the parameters linked to events (probability and value of the penalty) so that the sustainable strategy would yield the highest long-term payoff for the participants, while the myopic maximization the lowest one, with recovery loop strategies in the middle. Furthermore, we had to take into account that the policies are initially set up for maximizing production/consumption and can be only changed after the players’ decisions. Rounds of community council meetings are organized to discuss anything players would like to discuss. This is then followed by elections or referendum that determines which players serve as a governor and can make decisions for the subsequent round of policies: land (high or low land availability for agriculture, with respectively no or some areas allocated for rewilding that is expected to increase biodiversity in line with Pereira et al.^97^) and tax (high or low tax for households to cover the externalities, see also Carattini et al.^98^ for carbon taxes). The first round starts with policies maximizing production and consumption to increase ecological validity of contextualization. Therefore, in the first round, from the perspective of individual points it is optimal to maximize the payoff, only switching to a predefined strategy from the second round. Finally, as the game is planned for seven rounds it is also optimal for individual points to make the maximum decision in the final round. Payoff values represent expected payoff, taking into account penalties from the events weighted by the probability of event.

Based on the social dilemma, when all players follow the sustainable strategy, they should achieve the highest individual payoff by the end of the game, while short-term maximization strategy leads to the lowest payoff by the end of the game. The strategy that maximizes production and consumption when biodiversity is high provides an outcome similar to but slightly lower than the sustainable one. Still, it has to be emphasized that these results present the expected payoff, taking into account the probability of events and penalties linked to them. In a single game, the results may differ due to the probabilistic nature of these events.

As mentioned before, the first round has fixed decisions that generate a balanced market and a group payoff of 600. If players reduce production and consumption to a sustainable level, they can accumulate a payoff of up to 1968 in the long term. One-round results decrease but there is no significant risk of disasters. Myopic optimization results in an expected negative group payoff of −444, taking into account the probabilities of the penalties. Individual results may vary due to the imbalance in the market or the randomness of the disaster events.

### Pilots, analysis of debriefings, and reflections

**Fig. 9:**
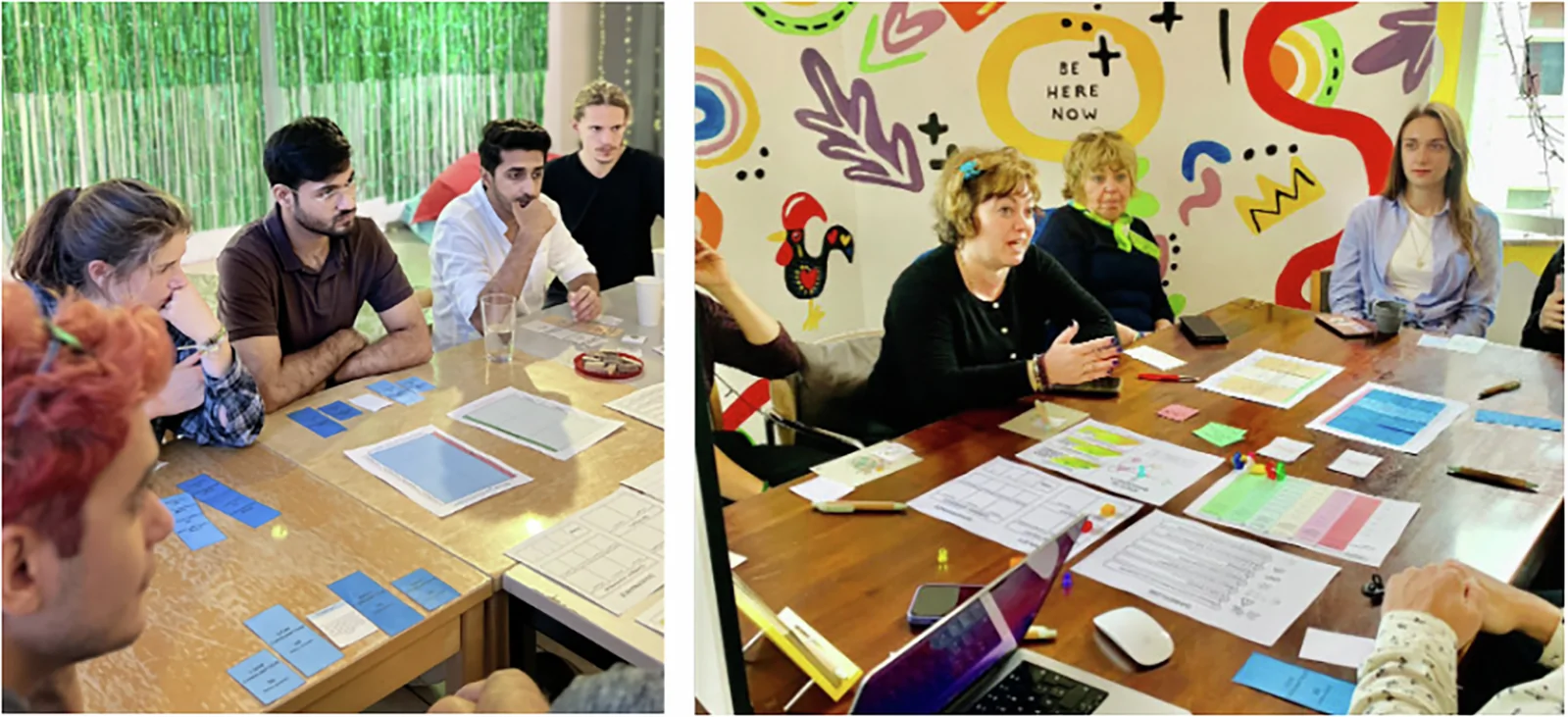
Moments captured from the game pilots with the project-related learning community members (left) and participants beyond the project (right).

The game was continuously pre-tested by the authors and their teams. The piloting followed a purposive sampling^99^, where we were interested to test the earlier versions of the game with specific audiences—we wanted to ensure it resonates with experts in biodiversity and participants without expertise in biodiversity. Here we report on the results of our in-depth content analysis of debriefing transcripts from the five pilots with a total of 50 participants conducted during the development of the game (more than one participant can play as one player). The pilots included (1) two groups of experts that represented the consortium partners with expertise on biodiversity-related topics in the context of different sectors (agriculture, education, trade, finance, fashion), intersectionality dimensions (age, gender, ethnicity, disability, religion), and geographic focus (Austria, Belgium, Brazil, Czech Republic, Germany, Hungary, Italy, Netherlands, Norway, Switzerland, UK) that met at the workshop conducted in Berlin on 18 January 2024 (*n* = 26; 16 female, 10 male; information on age was not collected, but it is a mixed and professionally-active age adult group of approximately 25–60); (2) sociology students studying structural change in a society as part of the course “SO2 Economy, State and Society” at the Martin Luther University Halle-Wittenberg that met in Halle on 2 June 2024 (*n* = 9; 5 female, 3 male, 1 non-binary, information on age was not collected, but it is a group of bachelor students mid-way through their studies in sociology and political sciences); and (3) a group of young people, including both prior participants in biodiversity-related activities of the learning community of the PLANET4B case study on urban youth and nature and newcomers primarily drawn by interest in the game itself that met in Erfurt on 9 September 2024 (*n* = 8; 1 female, 7 male; one 19-year old, four university students with migration background in their mid-20s, one international volunteer in their mid-20s, two young adults in their late-20s, one with family migration history and one unemployed after their bachelor studies; three of the participants played the earlier version of the game); and (4) an external group of audience that had no direct connection to the project, who attended the game piloting in Hamburg on 17 September 2024 (*n* = 7; 5 female, 2 male; information on age was not collected, one senior lecturer at a university in Hamburg, and a group of practitioners in the field of urban planning and architecture, two of whom were also students) (see the example photos in Fig. 9).

An iterative deductive and inductive analytical process was employed to explore patterns emerging from the qualitative data following Standards for Reporting Qualitative Research^100^. With the informed consents of the participants, the debriefings were recorded and transcribed using AI assisted transcription of sound and three rounds of subsequent manual correction. The transcripts were then analyzed using in-depth content analysis^99,101^, where initial codes were developed based on the theories discussed earlier beginning with open coding informed by close engagement with the transcripts, and iteratively adjusted based on the empirical evidence. Analysis was conducted using MAXQDA qualitative data analysis software. One researcher undertook the primary coding, and regular weekly meetings with two supervisors were conducted to enable collective reflection on emerging interpretations until we reached the theoretical saturation. An interpretive agreement was reached by first anchoring our initial codes in the discussed theories of social dilemmas. When the primary coder encountered participant statements that did not fit this deductive framework or shared critical feedback about the game’s premise and mechanics, these instances were treated as “critical cases”. These cases were actively sought out in subsequent iterations and brought to the weekly supervisory meetings, where the team collectively agreed on new, inductive codes (such as “affective-relational focus” or “frustration about the challenge”) to capture the added value from the empirical data. This iterative regrouping ensured that initially novel participant viewpoints drove the evolution of the coding framework rather than being forced into pre-existing categories. A framework with a hierarchy of codes (Table 1; see also the Supplementary Information for illustrative examples of quotations) evolved throughout the analysis, with new themes and sub-themes prompting iterative revisions and regrouping of codes to ensure alignment with the developing understanding of the data.

**Table 1:** The coding framework for debriefing analysis

Overarching categories	Definition and sub-categories
First-order social dilemma 1: Cognitive focus in social dilemma	This includes when a person mentions the challenge in the sense of individual versus group, I versus others, short versus long term, biodiversity versus food or similar.
First-order social dilemma 2: Affective-relational focus in social dilemma	This includes when a person mentions the challenge in the sense of trust in relations, identifying with the group, frustration with others or similar.
Second-order social dilemmas: Political and governance dilemmas, including intersectionality-related dilemmas	This includes when a person mentions power of the market, democracy and elections, intersectionality dimensions that explain systemic barriers and privileges, collective action, role of communication.
Lessons specific to tackling biodiversity loss	This includes when a person mentions complexity of the system, frustration about the challenge, hope or other emotions, other biodiversity-specific lessons such as probability events and resilience.
Game-reference and feedback	This includes when a person describes what they are referring to in the context of the game (e.g., game mechanics).
Real-life reference	This includes when a person describes what they are referring to in the context of or through comparisons with real life.

### Ethical statement

Ethical approval for this project was obtained from the German Association for Experimental Economic Research e.V. (Institutional Review Board Certificate No. Yuh2H9y1) and from the Ethics Commission of the Martin Luther University Halle-Wittenberg (Reference Approval No. 2308SIU). The authors have obtained written informed consent from the participants before joining the study as well as to publish images.

## Supplementary information

260907_Supplementary_Information_Pathbreak.

## Data Availability

The pseudonymized data from the debriefing sessions that were recorded, transcribed, and analyzed for this research is available at https://doi.org/10.5281/zenodo.17476103. The fully coded datasets are not publicly available due to notes that contain information that can lead to identification of individuals but are available from the corresponding author on reasonable request.
