# Supplementary material for: Pathbreak: A Biodiversity-Food-Governance Game for experiential reimagination of social-environmental futures
Source: NPJ Biodivers. 2026 Oct 8;5:35. doi: 10.1038/s44185-026-00160-7 (PMC13645720; doi:10.1038/s44185-026-00160-7)
Supplement: Supplementary file 1 [file 44185_2026_160_MOESM1_ESM.pdf]

# Supplementary Information to the Manuscript

## Pathbreak: A Biodiversity-Food-Governance Game for experiential reimagination of social-environmental futures

*Ilkhom Soliev [1,2,3,]*, Michał Pająk [2,4], Maryna Bykova [5], Marco A. Janssen [6]*

[1] Department of Environmental Sociology, Martin Luther University Halle-Wittenberg, Halle (Saale), Germany
[2] Social Learning and Environmental Governance (SLEG) Lab, Centre for Interdisciplinary Regional Studies (ZIRS), Martin Luther University Halle-Wittenberg, Halle (Saale), Germany
[3] German Centre for Integrative Biodiversity Research (iDiv), Leipzig, Germany
[4] Wroclaw University of Economics and Business, Wrocław, Poland
[5] Culture Goes Europe (CGE) Erfurt e.V., Erfurt, Germany
[6] Center for Behavior, Institutions and the Environment (CBIE), Arizona State University, USA

* Corresponding author: ilkhom.soliev@soziologie.uni-halle.de; ilkhom.soliev@idiv.de

**Table S1.** Codebook with illustrative quotations and coding explanation

| **Overarching categories** | **Definition and sub-categories** | **Illustrative quotations (including negative and ambiguous cases) and brief reasons** | **Remark on coding/reasoning** |
|---|---|---|---|
| First-order social dilemma 1: Cognitive focus in social dilemma | This includes when a person mentions the challenge in the sense of individual versus group, I versus others, short versus long term, biodiversity versus food or similar. | **Speaker**?: Yeah, I think so too. This is quite personal really. But I think that in my sort of game made me think about my own personal politics. And I think my own personal Politics is that you should make collective decisions. But in the UK at the minute, there's a very strong narrative for individualism. And in the game, I kind of felt reassured by ourselves that making a collective decision and making collective decisions about what is enough, about how you work together and you don't need to be much above zero, if zero was | The participant reflects on "personal politics" versus "collective decisions". |

| | | | |
|---|---|---|---|
| | | almost like food. Yeah, it was interesting in that sense that it made me think about that. [#00:07:23#] (1 Experts 1 (Berlin), Pos. 42) | |
| | | Person 1: But you're still dependent on the decisions of others. Yeah. Yeah. #00:01:48-0# (2 Experts 2 (Berlin), Pos. 19) | The participant reflects on dependence on others. |
| | | Person 5: Uh, because we're out to, uh, uh, government for a long period, and now we have a new government, and they're still doing a lot of good things, but still sitting on the old things that were, um… #00:18:53-6#<br><br>Person 1: Legacy from the previous. #00:18:54-9#<br><br>Person 5: And their legacy from the previous. And, yeah, the, the things that they are going to change might not even appear to any to everyone, um, when there's a new election next year, for example. So yeah, that's kind of just how long it takes to change things. #00:19:13-7# (3 Students (Halle), Pos. 67-69) | Participant reflects on short versus long term. |
| | | **Speaker**?: So, I was always confused about. What I think about the Consumer. So, should I think about myself? Uh, I'm one with, uh, Let's say politics and economy. I always think about people. That's why our… eh, because of I was alone, uh, Italy, uh, among the farmers, let's say, uh, I always wanted to like to | The participant reflects on food production and links to consumers, politics, economy.<br><br>Somewhat ambiguous link. |

| | | | |
|---|---|---|---|
| | | discuss with, uh, uh, you and you, but I couldn't. Uh, and so the last moment. So, when uh, ministers, like, put high, I thought maybe he needs more food. So, I had to increase production, and then... (Laughter) [#00:15:02#] | |
| First-order social dilemma 2: Affective-relational focus in social dilemma | This includes when a person mentions the challenge in the sense of trust in relations, identifying with the group, frustration with others or similar. | **Speaker**?: With the municipality people. But you need a lot of trust. Uh, I mean, people need to, you need to have a relationship that they are willing to play a game. This is nothing they usually would share with others to. I mean, it's even a bit low play to say like this. And, um, I also thought that it would need to have… I think it would be really good learning exercise to say that it is, uh, but it needs to have a format which is easily accessible for the people. So, uh, if, you know, I imagine my feeling, I think it took me ten minutes until I could get into it. And I was very much willing to go into it. If it has someone on the table not willing…Finished. [#00:45:48#] | The participant reflects on the role of trust to play the game with stakeholders.<br><br>Covers also the potential results from lack of trust. |
| | | Person?: But I think in that sense, it's quite interesting how, um, I think you would usually, I think, put low, uh, what is it? Consumption I think. Just because you don't want to stand out as much, maybe in a way of trying to fit in maybe. Um, because I think that happened now. I don't think you put a lot of high consumption cards. I think that's what I've noticed. Um, compared to others, in a | The participant reflects on fitting in and still being different from the group.<br><br>Somewhat ambiguous but it seems about identifying with the group. |

| | | | |
|---|---|---|---|
| | | way of what the actual situation was. So, um. Yeah. #00:28:50-3# | |
| | | Person 3: (...) I think it's quite difficult, like we saw to, please everyone's, um, demands on at some point, especially our motto to kids. So I think it's also in reality like that it's very difficult to. Yeah. Please everyone. #00:31:12-3# | The participant is frustrated and pleading to others. |
| | | And you gave like, the card, like, uh, first to the consumers and then to the farmer. So just kind of introduce a feeling of division to me, at least in the sense of the game. #00:17:57-3. | The participant reflects on the division between different roles created by the game role cards. |
| Second-order social dilemmas: Political and governance dilemmas, including intersectionality-related dilemmas | This includes when a person mentions power of the market, democracy and elections, intersectionality dimensions that explain systemic barriers and privileges, collective action, role of communication. | I'm not sure what I was thinking that. I mean, the governor has a lot of power, obviously, but he cannot control all of the formal actions, all of the actions from the business actors. In the end, I mean, they were farmers and they were like, um, kind of doing the same after she said, we're just going to put 40. Um, but yeah, that's obviously not going to work in society because people are always interested in their profit. | The participant reflects on the power of politics and actors interested in their profit. |
| | | (...) Yeah. as I said, for the legacy of a government will only show later. It's a good thing that people learn about that, I guess. And yeah, I mean, it's, um, equivalent to our situation in Germany as well. | The participant reflects on the time needed to effect change in democracy through elections. |
| | | **Speaker**?: (...) For me. Um, I think in terms of diversity and inclusion. The moment we said or the moment we were | The participant reflects on the role of the structured intersectionality dimension |

| | | | |
|---|---|---|---|
| | | told the migrant didn't have any voting rights. It for me, it almost put a stigma on that individual, which I think changed the dynamics immediately. And, and I just, you know, I think completely wrong, but I just sense the dynamics changed around the table in the sense that this person now has no rights. Um, I think it would be interesting if you had, depending on the number of players you have, if you have name and number or lot number, and then suddenly find that person not being able to have it changes everyone else's position. Does that make sense? [#00:57:20#] | introduced through the role of a migrant player. |
| | | Person 3: Concerning the team play again. I was just thinking why we did discuss so much on our side. Because we had the impact on the biodiversity sort of gameplay. I think that was the reason why we were talking so much in our field. I'm not sure the consumers had that kind of decision as a group to make, to make a decision for something. So in a way, I think that sort of convinced us to work together because we did only have an impact together, but the consumers didn't have that. So I think that was maybe one of the reasons why we did team play so much then. Um, maybe that's that's one thing, maybe that that there needs to be a thing that the consumers need to change in a group of sort of sends. Maybe that's a thing. To have that team play, but I'm not sure in what ways that could be | The participant reflects on the role of discussing in a group and group decision making. |

| | | | |
|---|---|---|---|
| | | implemented, or if that it would even be helpful. #00:24:13-0# | |
| | | Person 3: Um, so I think maybe like the next, um, game we should, uh, take more advantage of, like, talking together about the problems. So, it was just my my feeling. Yeah, but I was, like, always struggling. Okay. What should I do? Should I do this? Do that? What is good for my kids. And like, there was like a completely other discussion and when one part I was actually wondering if that was, uh, um, on your card or on your character that you're like, I don't know, just, um, talking about the citizenship. So I was like, is it, is for... #00:21:52-6# | The participant reflects on the role of communication and being political. |
| Lessons specific to tackling biodiversity loss | This includes when a person mentions complexity of the system, frustration about the challenge, hope or other emotions, other biodiversity-specific lessons such as probability events and resilience. | Person 4: I agree, I found it interesting, too. Um, yeah. And it was interesting how it's all connected. Um, and that you have to plan ahead. Um, to think, okay, how do I affect biodiversity? And but I still want money. Uh, as a farmer. Um, what about consumer, a, uh, one? Yeah. That's, uh, was quite interesting. #00:03:13-6# | The participant reflects on the interconnectedness of various elements – complexity – and trade-offs. |
| | | Person 2: Yeah, I feel a little bit hopeless. Um, on the side of the, um, consumers. Um, I mean, we do have a way of changing by diversity, which is selecting a politician that regulates, um, the farmers and then convinces the farmers to act together and not produce too much. Um, I was thinking about that. I don't I don't know if I keep adding too much complexity, unnecessary complexity to | The participant reflects on being frustrated/hopeless. |

| | | | |
|---|---|---|---|
| | | the game, but maybe for the consumers to have, the option to choose what type of farmer they're supporting. Because we have a farmer that is, for example, more economically, more ecologically, uh, oriented. Uh, so maybe for farmers to be able to, to support, uh, farmers, for consumers to support that farmer more, that meaning that that farmer has a bit more influence or for the other farmers to be like, oh, okay. So if the market works that way, then we could also orient ourselves more ecologically. That could give um, consumers a bit more of an influence, rather than just hoping that some someone announces themselves as a green politician. #00:50:58-3# | |
| | | Um, otherwise, I still think that the game has um, I mean, I don't know, I don't, I don't know the, the exact motive, the goal, but I think it does show that, um, what we do, um, affects biodiversity. And the way that the market works affects biodiversity and that it has negative consequences that we can try to solve. Um, and in a certain context, um, can we just need to be hopeful, but it can be eye opening for some people, and I think that's what matters. #00:13:08-3# | The participant reflects (optimistically) about the power players have and the hope. |
| | | Person 5: Well, yeah, as I said before, the cross, uh, cross thing and, um, also that the climate change is in, uh, in, um, it's connected to the biodiversity because of the catastrophes, um, that will come with | The participant reflects on the connection between biodiversity and resilience. |

| | | | |
|---|---|---|---|
| | | the climate change. The biodiversity will, um, go down. #00:47:54-0# | |
| Game-reference and feedback | This includes when a person describes what they are referring to in the context of the game (e.g. game mechanics). | Oh yeah. One one, one thing that also was suppressed that we I think it doesn't matter how we will start at this stage of reduced biodiversity after the first round anyways, because there are no limitations on land use. So already working on with that, um, framework from the first round on um, and then I mean, the game is kind of decides to, to act in that sort of like reaction way. Um, I don't know if it was intentional. | The participant reflects on the game mechanics, namely the possible impact from the sequence of rounds in the game. |
| Real-life reference | This includes when a person describes what they are referring to in the context of or through comparisons with real life. | Person 4: You could, uh, if you see this game as the whole planet all participants on the in the whole world. Yes, but I think it's more applicable to the Western countries like US, Canada and Europe and less to, let's say, Bolivia or something, because they do not have the structure. They produce a lot of stuff for actual for us, for Europe, for the US. So then again, we influence this with our behaviour. And they do not act that way. #00:59:32-7# | The participant reflects on the parallels with the real world. |

**Table S2.** Excerpts illustrating differences across participant groups

| |
|---|
| <u>Experts talking about the game as a tool and providing feedback on its design:</u><br>“There were some elements that, uh, frustrates me, which I struggle to connect with, let's say real life. Other than the fun, enjoy that comes with it. There's no question of this. But, for example, I question myself, why do we start from grief? Uh, because it's frustating that actually in real life we're not, realistically speaking, in a green area, right? It feels like we are in a showroom, right area somewhere. Um, and of course it does gives the all the way back gives the impression of a long road ahead to reach the green space, so to speak. But still, the frustration comes when we're not there. We told all the way to red and so on. So these elements gives me some frustration to connect with real life. Not again, not touching upon the fun part and then everything else that comes with it, which is also great. [#00:02:54#]”<br><br><u>Students talking about politics and governance, social dilemmas, and specific biodiversity lessons:</u><br>“Person 4: In one place. I think it's very difficult. I think of it. I think it's a systemic problem. Um, I thought about this issue we had, uh, recently protest of farmers, um, because they, uh, politics and limitations. We remember them. And then I thought it's difficult for farmers to. It's not that they make lots of tons of money and swimming it every day. Um, it can be hard for them, too. And I think we as consumers signal all the time that we want more and more and more. And all the supermarkets have to be full each and every day. Even at the end of the day, I want all the products. And yeah, and I think it's on every level. And I think we as consumers could start by thinking more about how we behave and do we really need to have all everything all the time? So only then the farmers can go back to, to uh, more, um, sustainable ways of producing, producing because they know they produce so much because there are some, there's so much, uh, need for that. #00:53:30-1#”<br><br>“If I had to say it's um, on the one hand, cooperation. Um, but also on the other hand was very, very dependent on, on the farmers. The farmers needed to agree. Uh, and the governor needed to agree whether we, uh, want to whether they want to protect biodiversity. And that was the main aspect of the game play, because, um, you can put taxes or you can consume more or less as a citizen or consumer. But at the end of the day, it was more about agreeing between the people. I mean, it showed that that improvement comes from agreement, uh, in between the people, among the people, that power. Because if the farmers don't agree to produce less, uh, we get to disaster after disaster. And if the governor does not put taxes or does not reduce the use of land, we also get into disaster after disaster. So that's one of the main things. And go through this okay. #00:08:58-0#”<br><br>“Person 2: Yeah. To get to the point, um, I think we would want to say so much point. it. Okay. Um. The thing is, the game shows the importance of biodiversity. And I think that was like, the most important point. And, I mean, most of the things were said. I really don't know what to add to it. Um, what? Uh, I also wanted to say what I liked was the extreme poverty of poverty and, um, this table. So you could, could see how your state is ranked. No. Not really. Not that it changed. But yeah, that was like a sort of thing. #00:10:42-8#” |

Learning community members talking more about key social dilemma:

"And the second thing is that individual people make their decisions. That affects biodiversity more than the political decisions. So if they are like choosing to be for personal greed and the personal greed or the point or something like that, it is it has more priority than then the biodiversity then the lesson is you're not going to win. You are going to eventually lose because, in the longer run you will face difficulties."

External group focusing on key social dilemma most, politics and governance of it, and lessons specific to tackling biodiversity loss:

"So my strategy to win this game was to stay in the Green Zone until the year 4 or 5 and that and then come up with an exit scam and to say to the voters that I would stay on a "low land availability", but after the election, I would go for "high land availability" and then go for 100 production for the last two, three rounds to win this game. But it's as I said before, it's also not working for someone who is profit-oriented because in the moment or to stay in the Green Zone, I'm also dependent on the other producers. And if they not choose 40 production, this strategy goes wrong."

"We have to be more proactive politically."

"This game inspired me so much, that I want to show it to others. That why I invited everyone for today's round. For me the game was... I think we don't talk much about biodiversity in the society, and this is why this game round is a such possibility for talking."
